# Immunosenescence: a key player in cancer development

**DOI:** 10.1186/s13045-020-00986-z

**Published:** 2020-11-10

**Authors:** Jingyao Lian, Ying Yue, Weina Yu, Yi Zhang

**Affiliations:** 1 Biotherapy Center and Cancer Center, The First Affiliated Hospital of Zhengzhou University, 1 Jianshe East Road, Zhengzhou, 450052 Henan China; 2 State Key Laboratory of Esophageal Cancer Prevention and Treatment, Zhengzhou, 450052 Henan China; 3 Clinical Laboratory, Henan Medical College Hospital Workers, Zhengzhou, 450000 Henan China

**Keywords:** Immunosenescence, Tumor progression, Aging, Tumor microenvironment, Cancer immunotherapy

## Abstract

Immunosenescence is a process of immune dysfunction that occurs with age and includes remodeling of lymphoid organs, leading to changes in the immune function of the elderly, which is closely related to the development of infections, autoimmune diseases, and malignant tumors. T cell–output decline is an important feature of immunosenescence as well as the production of senescence-associated secretory phenotype, increased glycolysis, and reactive oxygen species. Senescent T cells exhibit abnormal phenotypes, including downregulation of CD27, CD28, and upregulation of CD57, killer cell lectin-like receptor subfamily G, Tim-3, Tight, and cytotoxic T-lymphocyte-associated protein 4, which are tightly related to malignant tumors. The role of immunosenescence in tumors is sophisticated: the many factors involved include cAMP, glucose competition, and oncogenic stress in the tumor microenvironment, which can induce the senescence of T cells, macrophages, natural killer cells, and dendritic cells. Accordingly, these senescent immune cells could also affect tumor progression. In addition, the effect of immunosenescence on the response to immune checkpoint blocking antibody therapy so far is ambiguous due to the low participation of elderly cancer patients in clinical trials. Furthermore, many other senescence-related interventions could be possible with genetic and pharmacological methods, including mTOR inhibition, interleukin-7 recombination, and NAD^+^ activation. Overall, this review aims to highlight the characteristics of immunosenescence and its impact on malignant tumors and immunotherapy, especially the future directions of tumor treatment through senescence-focused strategies.

## Background

The morbidity and mortality rates of various tumors increase with age, and thus, malignant tumors are generally defined as aging diseases [1, 2]. It should be noted that aging is generally defined as a decline of function in living organisms that occurs in a time-dependent manner and is associated with cancer progression [3]. Despite many studies considering aging as a tumor-suppressor mechanism, most senescent cells behave abnormally, which may eventually lead to serious outcomes, such as the development of tumors. Moreover, the accumulation of DNA damage, a critical driver of senescence, and the concomitant events associated with cellular senescence have been shown to participate in tumorigenesis. These studies also documented that cellular senescence is a cellular state closely associated with various physiological processes and aging-related diseases [4, 5] and is a double-edged sword in cancer [6]. Nevertheless, small-scale cellular senescence does not represent systematic senescence: only when the scale of cellular senescence gradually increases and affects the whole system, senescent phenotypes and age-related diseases, such as malignant tumors, may occur [7].

The immune system has an ambiguous role in cancer, as it plays an important immune surveillance role in the antitumor response but is also closely associated with the initiation and progression of tumors [8]. Moreover, immune system aging, also known as immunosenescence, is a natural process that occurs with age and leads to a decline in immune function, thus affecting various aspects of immune functional networks and increasing cancer risk. The concept of immunosenescence was first proposed by Walford in 1964 [9] and is characterized by decreased adaptive immunity, decreased infection resistance, and increased autoimmune risk [10, 11]. In addition, a variety of factors can dramatically influence this status, such as genetics, exercise, nutrition, previous exposure to microorganisms, sex, and human cytomegalovirus infection [12–16]. It could therefore be possible to target the immune system of the elderly aiming to restore its competence [17]. However, the main obstacle to achieving efficacious immunotherapy is the tumor microenvironment (TME), which may accelerate senescence of the immune system [18, 19]: potential targets for rejuvenating the immune system are still a hypothesis. Many studies have shown that the tumor response of innate and adaptive immune systems is different between young and elderly individuals, but its clinical impact and underlying mechanisms are still mostly not understood. For example, T cells are the main effectors of acquired immunity, and their compartment is heavily affected during aging, cumulating defects [20] that can increase immune system damage, disease susceptibility, and the occurrence of malignant tumors in the elderly. Therefore, these lines of evidence indicate that the underlying mechanism of tumorigenesis is closely associated with immunosenescence.

Although our understanding of immunosenescence has steadily progressed over the past few decades and many studies on age-related immune decline have laid the foundation for identifying intervention methods [10, 21–23], the interactions between senescence-related changes and different components of the immune system remain unclear. Here, we discuss the most relevant strategies for current research on immunosenescence, focusing on its characteristics and on the various types of immune cells during aging, also highlighting the pivotal role of immunosenescence in tumor progression and immunotherapy. Moreover, we also discuss the interaction between cancer cells and the aging TME, and address how the latter drives tumor progression. Finally, we outline possible future immunosenescence-based interventions that may affect tumor progression.

## Concept, process, and hallmarks of immunosenescence

**Fig. 1:**
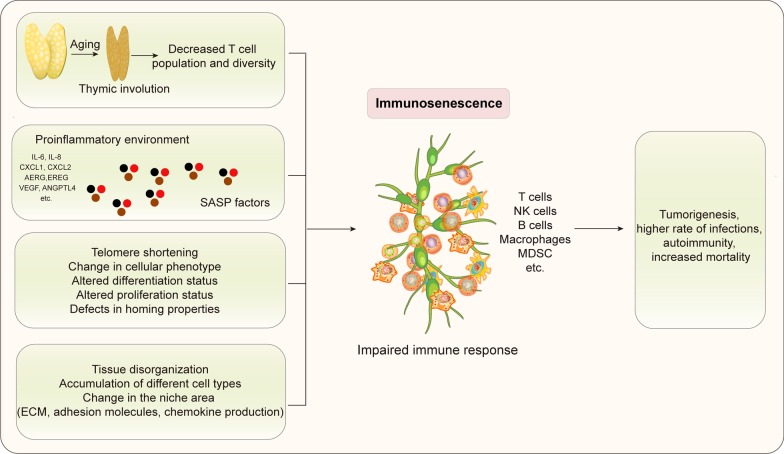
The process of immunosenescence. The process of immunosenescence can alter the immune response, thus leading to the occurrence of various diseases, such as tumors and infections. Many factors can activate the immunosenescence process: the thymus gradually degenerates, resulting in an age-related decrease in T cell output and leading to the senescence of the immune system; inflammation related to advanced age will produce SASP, which also leads to immunosenescence; the intrinsic factors in immune system cells as well as potential extrinsic factors that are often overlooked, can also cause immunosenescence

Senescence is a normal physiological process in which organ function slowly changes with age. When it concerns the immune system, it is termed immunosenescence [9]. As a consequence, immunosenescence is a process of immune dysfunction that occurs with age and includes remodeling of lymphoid organs, leading to changes in the immune function of the elderly. In fact, the process of immunosenescence is regulated by many factors, including aging, chronic inflammation, and changes in the microenvironment (Fig. 1). Moreover, an important distinguishing feature is that the thymus gradually recedes and degenerates with age, resulting in a decrease in T cell output [24, 25]. This process is considered to be a manifestation of immunosenescence. Therefore, understanding how to restore thymus function and the production of T cells provides an effective strategy for immunosenescence intervention. Additionally, inflammation related to advanced age will produce a senescence-associated secretory phenotype (SASP), which also leads to immune system senescence [15, 23, 25]. Furthermore, intrinsic factors in immune system cells [26–28] as well as potential extrinsic factors that are often overlooked can also cause immunosenescence [29]: its complexity supports the idea that it will lead to the damage of immune response, leading to the occurrence of various diseases.

**Fig. 2:**
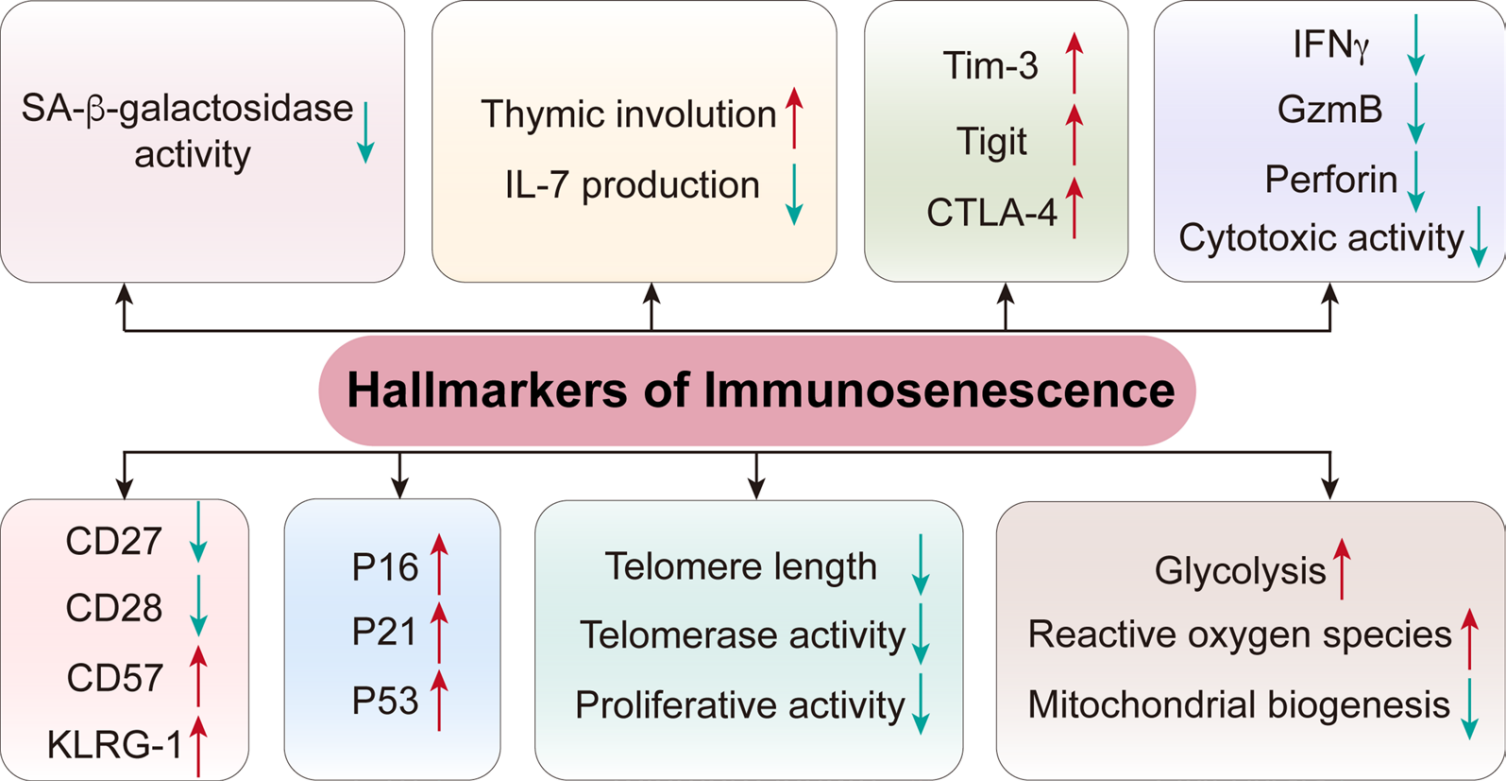
The main markers of immunosenescence. The main feature of immunosenescence is the degeneration of the thymus, which is accompanied by a decrease in IL-7 secretion. Senescence is accompanied by a decrease in telomere length and telomerase activity. Senescence T cells show reduced SA-β-galactosidase activity, cytotoxic activity, expression of functional molecules such as IFN-γ, scarce proliferation, and also arrested cell cycle, due to an increase of the related proteins, P16 and P21. During the remodeling of the immune system with age, changes in immune cell markers are mainly characterized by the loss of CD27 and CD28 expression and the increase in CD57 and KLRG-1 expression. During aging, glycolysis and ROS production are increased, while mitochondrial synthesis is decreased

Consistent with this concept, the identification of hallmarks and characteristics associated with immunosenescence is essential for exploring its impact and significance, especially on tumor progression (Table 1) (Fig. 2). Indeed, some potential targets were already investigated: a recent study has reported that interleukin (IL)-7 and its receptors play a key role in immunosenescence, affecting the balance of the immune system [30] (Fig. 2). More recent studies have shown that, during aging, the cytotoxic activity of immune cells decreases, and the expression of functional molecules associated with cytotoxic activity, such as interferon gamma (IFN-γ), granzyme B, and perforin, is also reduced [31–34] (Table 1). Additionally, as the immune system ages, metabolism also changes, showing increased glycolysis and reactive oxygen species (ROS) production, as well as decreased mitochondrial synthesis [35, 36]. It has been proposed that SASP, a unique characteristic of aging cells, wherein these cells secrete a variety of soluble factors, including growth factors, cytokines, proteases, chemokines, and extracellular matrix (ECM) components, mediates the paracrine activity of senescent cells, thus inducing several senescence-related diseases, including various malignancies [37].

**Table 1:** The main characteristics of immunosenescence cells

Category	Markers	Cell types		References
Surface marker	CD27, CD28	T cells	↓	[41, 42]
	CD57, KLRG-1, Tim-3, TIGIT, CD45RA	T cells	↑	[41, 42]
	NKp30, NKp46, DNAM-1, NKG2A	NK cells	↓	[28, 45, 46]
	KIR, NKG2C, CD57	NK cells	↑	[28, 45, 46]
	CD62L, TLR1/4	Monocytes/macrophages	↓	[28]
	CD11b, TLR5	Monocytes/macrophages	↑	[28]
	CD11a, CD11b	Neutrophils	–	[28]
Cell cycle arrest	P16, P21, P53	T cells	↓	[5, 42, 43]
Molecules associated with DNA damage	γH2AX	All	↑	[64, 65]
TCR signaling machinery	Lck, ZAP70, DLG1, Lat, SLP-76	T cells	↓	[39, 64]
Proinflammatory cytokines	IL-6, IL-8, IFN-γ, TNF	T cells	↑	[31–34]
Cytokines	IL-7	T cells	↓	[30]
Inhibitory factors	IL-10, TGF-β	T cells	↑	[39]
Epigenetic change	SAHF	T cells	↓	[37]
Metabolic alteration	Glycolysis	T cells	↑	[35, 36]
	Mitochondrial biogenesis	T cells	↓	[35, 36]
	ROS	T cells	↑	[35, 36]
Effector molecules	Perforin, GzmB	T cells	↓	[39]
Telomere length	Telomere	All	↑	[21, 44]
Telomerase activity	Telomerase	All	↓	[21, 44]
SA-b-gal activity	SA-β-gal	All	↑	[34]

It has been well documented that the thymus is a lymphoid organ where T cells differentiate, develop, and mature. Thymus degeneration leads to a decrease in the number and proportion of CD8^+^ naïve T cells, which is one of the main manifestations of immunosenescence [24, 25] (Fig. 2). It is therefore likely that T cells are important immune cells and play a key role in the occurrence and progression of tumors. It is well known that senescence, anergy, and exhaustion are three important dysfunction states of T cells in cancer, and they are significantly different in terms of molecular regulation during tumor progression. T cell senescence is irreversible, as opposed to their anergy and exhaustion, both of which are considered reversible [31, 38–40]. Age-related immune biomarkers, such as the downregulation of the costimulatory molecule CD28 of senescent T cells, have also been employed in several senescence studies [41], which do not express costimulatory molecules such as CD27 and CD28 but express the killer cell lectin-like receptor subfamily G (KLRG-1) and CD57. Therefore, the T cell phenotype of CD27^−^CD28^−^CD57^+^KLRG-1^+^ is also an indicator of immunosenescence. Moreover, with the loss of CD27 and CD28, there is an increase in the expression of P16 and P21, which are involved in cell cycle regulation [42] (Fig. 2). P53 can cause cellular senescence [42] by regulating the cell cycle via inhibition of cyclin-dependent kinase (CDK) 4 and CDK6 [5, 43] (Fig. 2). Moreover, levels of immune checkpoint-related molecules, such as Tim-3, Tight, and cytotoxic T-lymphocyte-associated protein 4 (CTLA-4), also increase during aging (Table 1). Another hallmark of senescent T cells is telomere shortening [21, 44] (Fig. 2), which is caused by the continuous replication of T cells and the decrease in expression of the human telomerase RNA component. It should be noted that, in the process of immunosenescence, the activating receptor expression of natural killer (NK) cell markers NKP30, NKP46, etc., is reduced, while the inhibitory receptor expression of KIR, NKG2C, etc., is increased [45, 46]. With an increase in age, the expression of CD62L and TLR1/4 on monocytes and macrophages is decreased, and the expression of CD11b and TLR5 is increased [28]. However, the expression of CD11a and CD11b in neutrophils did not change significantly [28] (Table 1). Recent studies have identified new types of immunosenescence biomarkers, including circular RNAs (e.g.,*, circular RNA100783*) and microRNAs, e.g., *MiR-181a*, which is reported to be a T cell-specific senescence marker [47, 48].

## Immunosenescence and cancer

Generally, the risk of malignant tumors increases with age. In addition to the accumulation of genetic mutations, many researchers believe that immunosenescence may also play an important role in the tumoral process. DeSantis et al. [49] found a higher risk of tumorigenesis in the elderly group. By analyzing the age distributions of 100 different tumors, Palmer et al. [24] concluded that the immune system may play an important role in tumor development. Vatter et al. [50] performed a phenotypic analysis of mammary epithelial cells from 57 women (16–91 years old) and showed that accumulation of lumen and progenitor cells with age may lead to an increased risk of carcinogenesis.

**Fig. 3:**
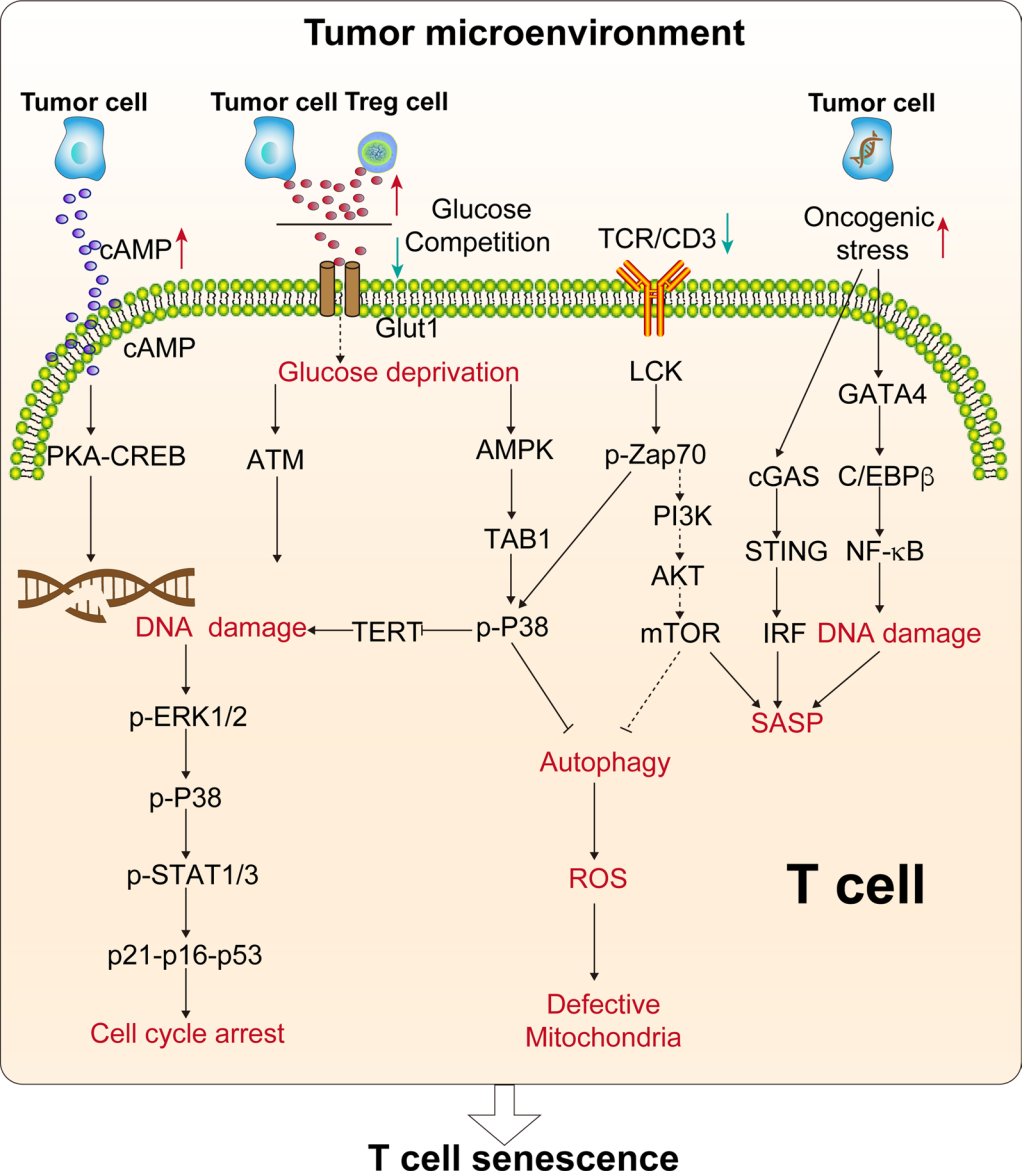
T cell senescence-related signaling pathways. Tumor-derived cAMP can be transferred to T cells, activating the PKA-CREB signaling pathway, which in turn activates DNA damage and induces T cell senescence. T cell competition for glucose triggers ATM-related DNA damage, activates the ERK1/2 and P38 pathways, and interacts with STAT1/3, which leads to T cell cycle arrest and aging. Activation of the P38 pathway induces the downregulation of TERT, leading to DNA damage. The downregulation of TCR signaling can activate the P38 pathway and inhibit the PI3K-AKT-mTOR signaling pathway, thereby inactivating autophagy and inducing mitochondrial dysfunction and ROS production in senescent T cells. DNA damage induced by the GATA4-C/EBPβ signaling pathway leads to increased secretion of SASP molecules. Similarly, the cGAS-STING signaling pathway also leads to increased secretion of SASP molecules

Many factors in the TME can induce the senescence of immune cells and dramatically affect their function. Multiple studies have shown that an immune system disorder in the elderly may promote tumor growth and induce T cell senescence, such as an increase in the proportion of tumor-associated macrophages and regulatory T cells (Tregs) [51–55]. The molecules and pathways associated with T cell senescence in the TME are shown in Fig. 3 [39]. Several studies have reported that tumor-derived endogenous cyclic adenosine monophosphate (cAMP) induces T cell senescence by inhibiting tumor-specific effector T cells in hypoxic microenvironment [56, 57]. In addition, tumor cells activate the PKA-CREB and P38 pathways by producing cAMP, thereby causing DNA damage and senescence of T cells [51, 53, 58–63]. Similarly, glucose competition triggers ATM-related DNA damage, activates the ERK1/2 and P38 pathways, and cooperates with STAT1/3, leading to T cell cycle arrest and aging. The activation of ATM and AMPK pathways induced by glucose can also cause DNA damage and T cell senescence, in which AMPK activates P38 by binding to TAB1 protein and downregulating the telomerase reverse transcriptase gene [64, 65]. P38 inhibits cell cycle progression by activating P53, P21, and P16 [64]. Moreover, aging T cells preferentially employ anaerobic glycolysis to generate energy, which results in mitochondrial dysfunction and increased reactive oxygen species (ROS) generation [36]. The NFκB, C/EBPβ, and cGAS-STING pathways also play a key role in the induction of T cell senescence [66]. Furthermore, the activation of Toll-like receptor 8 signaling in tumor cells can enhance antitumor immunity by blocking the induction of senescent T cells and tumor-specific T cells in vitro and in vivo and reversing their inhibitory effect [63].

In addition to basic research, several preclinical and clinical studies have been conducted on immunosenescence. Studies have shown that immunosenescence, especially that of CD8^+^ T cells, plays a pivotal role in the pathogenesis and treatment of patients with breast cancer [67]. Decreased expression of IFN signaling in CD8^+^ T cells in aged mice was identified in a mouse model of breast cancer [68]. The senescent microenvironment is closely associated with tumor metastasis and invasion, and the metabolism of T cells in the TME also changes with age. Compared with young patients with melanoma, ECM rearrangement resulting from Hyaluronan And Proteoglycan Link Protein 1 changes in elderly patients can cause melanoma cells to be more invasive and prone to distant metastasis [69]. The increased age-related sFRP2 secreted by fibroblasts also promoted angiogenesis and metastasis of melanoma cells [70]. In contrast to the above findings, senescence was also found to improve cancer prognosis. In various mouse models, tumor proliferation was found to be slower in elderly mice, such as MC38, B16, and 4T1 tumor models [71]. In 1973, a retrospective study of 226 patients with colorectal cancer aged 80 years and older found that the prognosis of elderly patients was better than that of young patients [72]. Older patients with bronchial cancer have slower tumor growth and reduced metastasis, which is believed to be due to the host aging factors that hinder the growth and spread of aggressive tumors [73]. In the elderly group of Engelbreth-Holm-Swarm cancer and B16F10 melanoma mouse models, tumor growth and metastasis sizes were lower and the subjects had a higher survival rate [74, 75]. Another study retrospectively analyzed the histological report of 1869 women with breast cancer and found that the incidence of invasive ductal carcinoma increased in patients < 39 years of age [76]. Recent research has highlighted that the upregulated methylmalonic acid in the serum of the elderly induces the expression of SOX4 and thus triggers the ability of transcriptional reprogramming, which can make cancer cells more aggressive [77]. Overall, these different research results indicate that age is closely associated with tumorigenesis.

Chemotherapy can also induce CD8^+^ T cell senescence in patients with breast cancer [67]. In a preclinical mouse model of pancreatic ductal adenocarcinoma, T/P drugs induced senescence of pancreatic cancer cells and activated SASP-dependent vascular remodeling, which not only enhances the uptake and efficacy of chemotherapy drugs but also promotes the infiltration of T cells into tumor tissues [78]. Altogether, these findings indicate that immunosenescence is linked to the occurrence and progression of tumors.

## Changes in senescence-related immune cell subsets in the TME

**Fig. 4:**
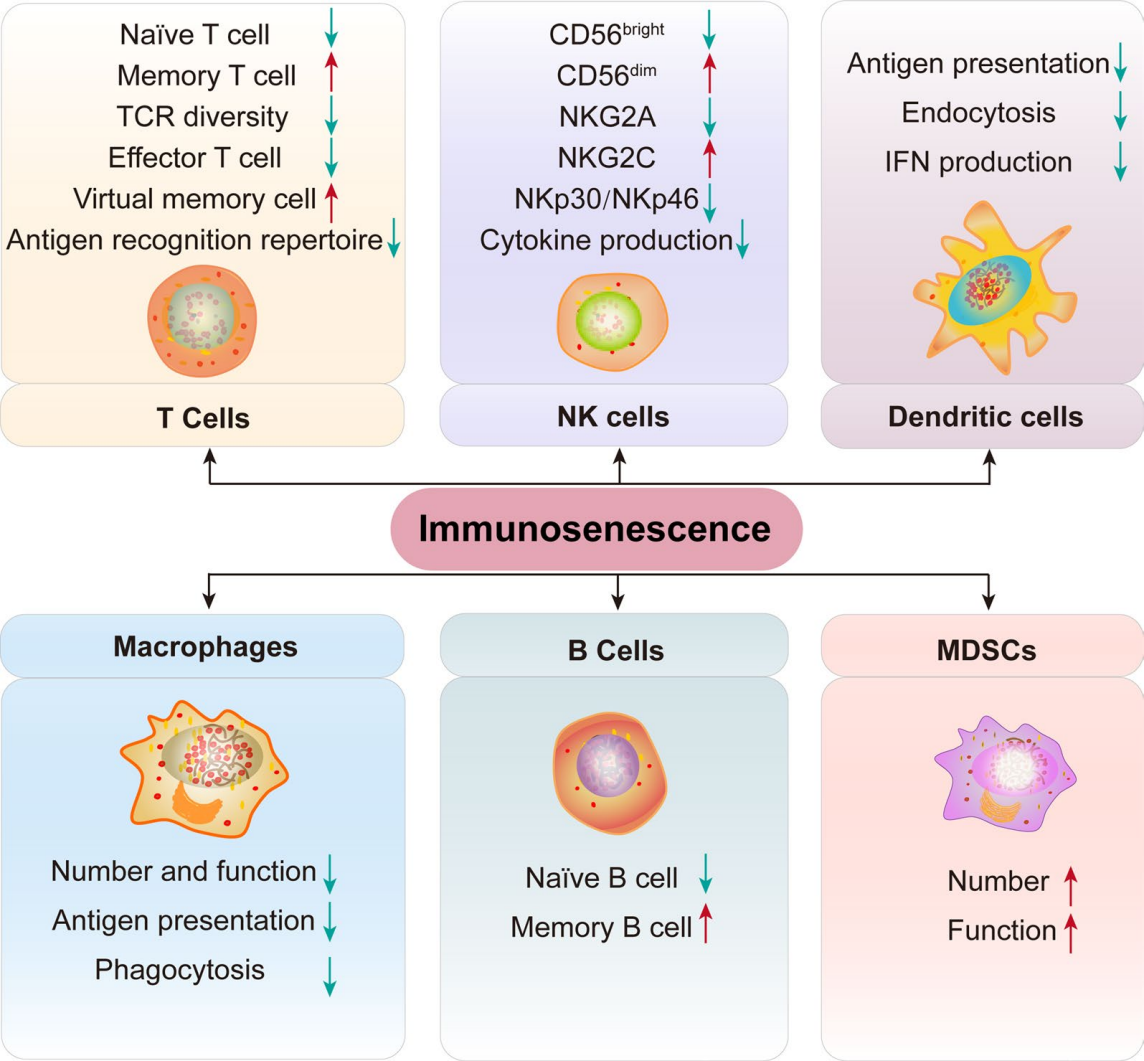
Changes in various immune cell subsets during immunosenescence. Many immune cell subpopulations are altered during immunosenescence. Number of both naive T and B cells is decreased, but the number of memory T and B cells is increased. In the aging process, the diversity of TCR decreases with age. In addition, antigen recognition and presentation capabilities are reduced. In addition, the antigen presentation and phagocytosis ability of DC cells are reduced. NK cell function is reduced. The number and function of MDSCs and macrophages are increased

Immunosenescence not only affects the innate immune system, which includes NK cells and macrophages, but also reduces the function of the acquired immune system, including T cells and B cells (Fig. 4). Thus, immune system senescence reflects the senescence of various immune cell subpopulations.

### NK cells

NK cells are a key component of innate immunity and, to a large extent, promote the antitumor immune response [79, 80]. The function of NK cells, such as immune surveillance, is also affected by age-related changes. The number of mature NK cells in all lymphoid organs and the expression of T-box protein, expressed in T cells (also called TBX21), and eomesodermin were both significantly reduced in aged mice [81]. Two subpopulations of NK cells, immature CD14^+^CD56^dim^ and mature CD14^+^CD56^bright^, are associated with aging and remodeling of mature NK cell subsets, and the reduced expression of activated receptors may promote the escape of NK cells in the elderly [45]. Moreover, NKp30, NKp46, and DNAM-1 are NK activation receptors involved in the recognition and lysis of different tumors, such as hematological malignancies [82], melanoma [83], and ovarian cancer [84]. Therefore, changes in the expression of these activated receptors in the elderly may affect the immune monitoring function of NK cells [85, 86]. The NK cell compartments in elderly patients with acute myeloid leukemia (AML) are also remodeled, as evidenced by the reduced output of immature CD56^bright^ cells and the accumulation of highly differentiated CD56^dim^ NK cells, which is associated with disease progression and survival [81, 87, 88]. Moreover, a clinical trial (NCT00799799) in which NK cells mismatched by KIR ligands were infused after immunosuppressive chemotherapy in elderly patients with AML showed that NK cell transfer is safe and feasible in elderly patients [89].

### T cells

#### Naïve T cells

Naïve T cells are a very relevant factor in immunosenescence research [90]: their generation is completely dependent on the thymus function, so the degeneration of the thymus is of great significance to the study of human immunosenescence [91, 92]. Degeneration of the thymus leads to structural changes and declines in function, which ultimately leads to a significant reduction in the thymus output of naïve T cells [93–95], thereby reducing the T cell antigen receptor diversity pool and ultimately leading to the destruction of T cell homeostasis. Therefore, degeneration of the thymus is the cause of age-related failure of the adaptive immune system [95, 96].

#### Memory T cells

Some studies also documented that memory T cells gradually accumulate with age [66, 97–99]. Memory T cells with a naïve phenotype also accumulate with age [100], and the number and ratio of memory CD8^+^ T cells in the elderly are usually higher, affecting their immune function. Collectively, these studies have demonstrated that central memory CD8^+^ T cells represent the majority of memory CD8^+^ T cells in aged mice [101, 102]. These virtual memory CD8^+^ T cells accumulate and exhibit characteristics consistent with aging.

#### Stem cell memory T cells (TSCM)

TSCM represent a small group of memory T cells with enhanced proliferation and differentiation characteristics, and they can produce more terminally differentiated daughter cells expressing effector molecules, which is of great significance for cancer immunotherapy [103, 104]. A study showed that there was no difference in the frequency of TSCM between the young and old groups [105]. Graham et al. also found that age significantly affected the distribution of other T cell subsets, but does not affect the frequency of TSCM [106]. Similarly, a study has shown that TSCM in young and elderly subjects is maintained through continuous proliferation and shows limited telomere length erosion and high expression of telomerase and Ki67 [107]. Overall, these results indicate that TSCM exists throughout the life process. However, a study has shown that the frequency of CD4^+^ and CD8^+^ TSCM is relatively stable at different ages, and the absolute number of CD8^+^ TSCM decreases with age [108]. Anis et al. found that aging is related to the loss of Wnt/β-catenin signaling in CD4^+^ TSCM and the increase of Dickkopf-related protein 1 (an inhibitor of the Wnt/β-catenin pathway). This study suggested that targeting Wnt/β could reverse the defect of TSCM through the catenin pathway, which may be a feasible method to restore and maintain immune homeostasis [109]. These results indicate that it is possible to further optimize culture conditions to obtain T cells with stem cell-like properties more efficiently, thus being able to further maintain and improve cancer immunotherapy.

#### CAR-T cells

Chimeric antigen receptor T (CAR-T) cell therapy is being developed as a potential treatment for patients with advanced hematological malignancies [110–116]. It has been reported that CAR-T cell therapy shows high response rates to B-ALL patients in different age groups [117–119]. However, a global study on CAR-T cells showed that this therapy is very effective on young patients with recurrent B-ALL [120], but did not discuss the impact on elderly patients, maybe due to the negative effect of the aging microenvironment. Moreover, CAR-T cell therapy is highly dependent on functionally active T cells, so T cell senescence plays a key role in immunosuppression and evasion of hematological tumors and solid tumors [121], underlying some practical challenges in this approach: the reconstructed and expanded CAR-T cells are exposed to the patient's tumor microenvironment, thereby inducing the senescent phenotype of CAR-T [122, 123]. Gabriele et al. reported that CD57, a T cell senescence marker, can quickly and efficiently transfer from glioblastoma stem cells to CAR-T cells, leading to its senescence [124]. Moreover, they also found that patient-derived glioblastoma stem cells were killed by CD133-specific CAR-T cells, but at the same time induced the expression of CD57, thereby inducing CAR-T senescence [125]. Furthermore, when CAR is introduced into T cells with unique T cell receptor (TCR) specificity, the presence of TCR antigen causes the loss of CD8^+^ CAR-T cell potency, which is related to T cell senescence, exhaustion, and apoptosis [126]. Moreover, recent studies have shown that uPAR-specific CAR-T cells can effectively eliminate senescent cells in vitro and in vivo and prolong the survival of lung adenocarcinoma mice [127]. These findings do suggest therapeutic potential of CAR-T cells for senescence-related diseases. In general, restoring the function of senescent CAR-T cells is the key to enhancing the antitumor effect of modified T cells.

#### ***CD4***^+^***T cells***

The low survival rate of patients with glioblastoma is associated with immunosenescence of postoperative CD4^+^ T cells [128]. The accumulation of the programmed cell death protein 1 (PD-1)^+^ memory phenotype CD4^+^ T cell subsets gradually increases with age and is the predominant subset in the aging phase of normal mice; this process is strongly accelerated during leukemia [129]. It has been reported that CD39 expression during aging reduces the number of CD4^+^ T cells [130]. Moreover, Tregs gradually accumulate with age [131–133] and the percentage of CD4^+^CD25^+^ Tregs in the peripheral blood of elderly mice significantly increased [134]. Similarly, in Lewis lung cancer mouse models and patients with lung cancer, Tregs infiltration and Foxp3 mRNA expression levels were higher in the elderly group than in the younger group [135, 136].

### B cells

After differentiation into plasma cells, B cells are the sole producer of antibodies and play a unique role in immunity [137]. The age-related remodeling of the B cell compartment may be the result of unbalanced fate selection in the hematopoietic progenitor cell population, autonomous changes in B cells, and external signals [138, 139]. The aging process leads to changes in the distribution of mature B cell subsets and impaired activation after stimulation [23, 140]. With increasing age, the proportion and number of CD19^+^ B cells in peripheral blood decrease and the function of B cells is impaired, which is reflected in the decreased expression of the autoimmune regulator AIRE and autoantigen genes in thymic B cells [141, 142]. In mice, the turnover of mature spleen B cells also decreases with age [143, 144].

### Other cells

**Fig. 5:**
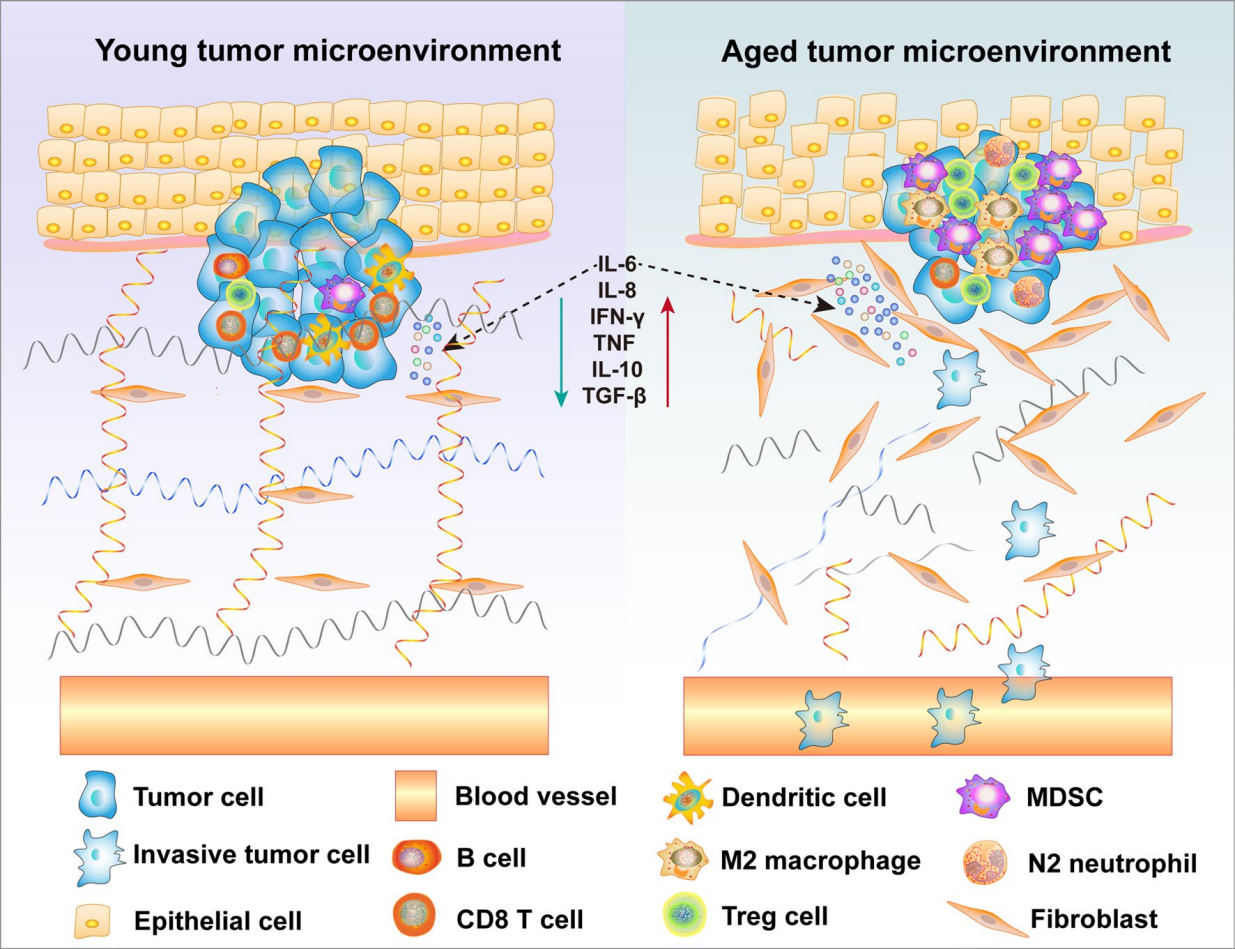
The effect of an aging TME on tumor progression. Age-induced changes in the structure and function of the ECM promote the occurrence and development of tumors. The integrity of the ECM is greatly reduced with age, which can promote the development of cancer. In the aging TME, stromal cells are disordered and loosely arranged, which may also lead to tumor progression and metastasis. Moreover, age-induced secretion of SASP, such as the accumulation of several factors, including IL-6, IL-8, and IL-10, is a key factor in inducing tumorigenesis and progression. The aging TME also exhibits an infiltration of immunosuppressive cells, such as MDSCs and Tregs. The increase in immunosuppressive M2 macrophages and N2 neutrophils may further promote immunosuppression, while immunosenescence of effector T cells, NK cells, macrophages, and DCs significantly reduces their cytotoxic activity, leading to tumor escape and tumor progression

Dendritic cells are the central coordinators of immune response and play a key role in immunity and maintenance of tolerance. Moreover, their functions such as antigen presentation, endocytosis, and IFN production, are also reduced in aged individuals [145, 146]. The ability of neutrophils to phagocytose pathogens decreases with age [147]. The phagocytosis and antigen presentation ability of macrophages change with age is not clear [147]. Age-modified tissue-specific macrophages and neutrophils may cause chronic low-grade inflammation, which is related to macrophage-mediated immunosuppressive disorders, together leading to the development of many diseases, including cancer [147]. Furthermore, increased levels of myeloid-derived suppressor cells (MDSCs) are observed in the bone marrow, blood, and spleen of tumor-bearing aged mice; this accumulation plays a detrimental role because MDSC-induced immunosuppression impairs senescence and tumor cell clearance and interferes with energy metabolism and maintenance of tissue protein homeostasis [58]. SASP-expressing senescent cells secrete several kinds of chemokines and cytokines, which recruit MDSCs to local regions and result in immune escape and tumor cell metastasis [58]. He et al. [148] found that in the first few weeks of mice and humans’ development, MDSCs can suppress T cells, and thus the accumulation of MDSCs is considered to result from a pathological process or pregnancy. The expansion of MDSCs may not only promote immunosenescence but may also induce harmful age-related bystander effects in host tissues by secreting TGF-β and IL-10 [149]. As shown in Fig. 5, the loose arrangement of the ECM in the aging microenvironment may be one of the causes of metastasis in elderly patients with tumors [69, 70]. Furthermore, in aging TME, changes in immune cell function and secretion of SASP molecules dramatically affect tumor progression [18]. Therefore, it is important to note the changes in senescence-related immune cell subsets in cancer research.

## Immunosenescence and tumor immunotherapy

Aging is a paramount risk factor for most cancers. Although the number of elderly people with cancer has increased dramatically, their inclusion in clinical studies is usually insufficient, as patients over 75 years of age represent less than 10% of patients with cancer [150, 151]. This hampers the provision of optimal medical services to the growing population of elderly patients with cancer. Nevertheless, novel cancer treatments targeting elderly patients are being prioritized due to the increasing aging population. Immunotherapy in malignant tumor treatment has dramatically changed the prospects of cancer treatment and improved the survival of patients with cancer. However, the results of immunotherapy in elderly and young patients are inconsistent, which is likely due to different types, different stages of tumor development, and concomitant diseases. Moreover, the association between immune-related adverse events and age is controversial, and thus analyzing the latest data on the efficacy of immunotherapy in elderly patients with cancer is paramount.

Immunosenescence may affect the response to blocking antibody therapy in patients with non-small cell lung cancer [152]. Importantly, a preclinical study highlights that the efficacy is poorer in older mouse models than in younger mouse models, indicating that age may have a significant effect on different tumor types and various physiological states [153]. Immune checkpoint blocking (ICB) antibody therapy has been used to treat a variety of tumors. Although age is one of the greatest risk factors for tumorigenesis, studies and clinical trials on the effects of host age on the outcome of ICB are limited and the influence of the microenvironment of the elderly on the response to immunotherapy is largely ignored. Little is known about ICB general tolerance and effect in the elderly. One study reported that age limits ICB antibody therapy efficacy in aged triple-negative breast cancer-bearing mice [68]. Kugel et al. [154] analyzed the relationship between age and anti-PD-1 response in 538 patients with melanoma and found that those over 60 years of age exhibited a better response to anti-PD-1 than those aged < 60 years. Elderly patients have also been observed to safely tolerate anti-PD-1/programmed cell death 1 ligand 1 (PD-L1) antibody treatment, with efficacy and toxicity effects similar to those observed in younger patients. A meta-analysis of randomized trials showed that although anti-PD-1 drugs are effective for both young and elderly patients, the risk of death among elderly patients is comparable or improved compared to that of patients subjected to control interventions, with no increased toxicity [155–158]. Although the toxicity in elderly patients was not increased, patients who experienced lethal toxic effects appeared to be older than those not suffering from lethal toxic effects [159]. Padron et al. [153] found that in a melanoma mouse model, anti-CTLA-4, anti-PD-1, and anti-PD-L1 antibodies were very effective in treating young mice. However, in the aged group, anti-PD-L1 treatment had no effect, whereas anti-CTLA-4 and anti-PD-1 treatment showed a positive effect. Another retrospective study indicated that anti-PD1 antibodies appear more effective and well tolerated in elderly (≥ 75 years old) patients with advanced melanoma than in younger patients [160], but these experiments did not conduct strict age-treatment subgroup analysis. Moreover, in an oral cancer tumor-bearing mouse model, the tumor regression of aged mice after anti-PD-L1 treatment was faster than that of young mice [161]. Furthermore, IFN signaling and antigen presentation were reduced in the TME of aged mice and patients with triple-negative breast cancer [68].

It is clear that more studies considering the high incidence of tumors in the elderly are needed, and that it is important to investigate the effect of ICB antibody therapy and its associated mechanisms in the elderly. In most studies, the number of elderly patients in the ICB antibody therapy group is small and data on the safety and toxicity of ICB antibody treatment are limited; therefore, the effect of ICB antibody therapy on elderly patients has not been fully elucidated [162]. Exploring the underlying mechanisms accelerating tumor growth and affecting immunotherapy in elderly patients is a challenging and urgent need. Therefore, further studies and clinical experiments are required to carefully evaluate the impact of ICB antibody therapy on the elderly.

## Clinical implications, evaluations, and intervention

The clearest evidence of age being the biggest risk factor for age-related diseases, and the accumulation of senescent cells is closely related to the occurrence of many chronic diseases [163, 164]. It has been reported that the reduction of senescent cells through genetic and pharmacological methods can prevent and alleviate various senescence-related diseases [165, 166]. Therefore, it will be critical to further understand how to test these interventions in humans and accelerate their widespread use (Table 2).

**Table 2:** List of the current clinical trials or drugs that are targeting immunosenescence

Treatment	Clinical trial/drugs	Target	References
Third-gen CAR-T cells containing CD28 + CD137	NCT02186860	CD28	[41]
Second-gen CMV-selected CAR-T cells against HER2 containing CD28.zeta signaling domain	NCT01109095	CD28	[41]
TAB08	NCT01990157	CD28	[41]
IL-7	IL-7	Thymic	[19]
KGF (keratinocyte growth factor)	KGF	Thymic	[19]
IL-22	IL-22	Thymic	[19]
Ghrelin	Ghrelin	Thymic	[19]
CDK4 inhibitors	CDK4 inhibitors	CDK4	[166]
Rapamycin	Rapamycin	MTOR	[166]
Metformin	Metformin	Mitochondrial respiration	[166]
NAD precursors	NAM\NR	NAD metabolism	[166]
Sirtuin-activating compounds	Resveratrol or trans-resveratrol\SRT2104\nicotinamide riboside	Sirtuin	[212]

In 1939, a study found that limiting the caloric intake of mice and rats could prolong lifespan, which proved for the first time that the aging process is plastic [167]. Some follow-up studies also found the same phenomenon in primates [168, 169]. It is worth noting that dietary restrictions not only increase lifespan but also inhibit the development of age-related diseases [170]. These findings further support the notion that life extension is related to aging and increased healthy lifespan.

One of the main characteristics of aging is the involution of the thymus, which leads to a decrease in the production of T cells, leading to immunosenescence. It has been found that there are many different ways to restore the structure and function of the aging thymus. Typically, a study has found that replenishing young transplantable thymic epithelial cells to middle-aged or defective thymuses can lead to increased thymus growth and T cell production, which directly drives the growth of degenerated thymus [171]. Similarly, a report has shown that the injection of a plasmid vector carrying FOXN1-cDNA into the thymus of elderly mice would cause a partial rescue in the size of the thymus and the number of thymocytes [172]. Therefore, targeted FOXN1 gene therapy may also be a great hope for revitalizing the structure and function of the aging thymus. IL-7 targeting the thymus of the elderly may also restore the development of T cells in the elderly. In line with this, it was reported that the IL-7 fusion protein, that is, IL-7 binding to the N-terminal extracellular domain of CCR9, could restore the thymus structure in elderly individuals [173], showing that targeted cytokine therapy has broad prospects (Table 2). Studies have also shown the relationship between physical exercise and improvement of thymic function in elderly patients: individuals who maintained physical exercise showed a slowdown in thymic output decline and changes in inflammatory markers, such as a decrease of IL-6 and an increase of IL-7 and IL-15 in serum, which may promote thymus function [174]. The use of thymus-targeted cytokines may be beneficial, but caution is needed because of adverse effects. Moreover, the development of effective interventions for age-related thymic degeneration requires further investigation.

Short telomeres can also trigger age-related diseases and shorten the lifespan of mice and humans. By activating telomerase to avoid telomere shortening, mice were seen to have longer telomeres, less DNA damage and aging, and extended lifespan [175].

Recently, it has been proven that drug intervention can slow down the aging phenotype to alleviate age-related functional decline [176]. Metformin, a widely prescribed antidiabetic drug, has been shown to alleviate aging in preclinical studies [177]. Positive effects on aging were also observed in mice, but these studies were carried out in short-lived mouse models that were prone to develop cancer in some cases [178]. In subsequent recent studies, in the relatively long-lived C57BL/6 mice and genetic hybrid mice, a similar phenomenon was observed [179, 180]. Furthermore, a retrospective analysis showed that the life span of diabetic patients treated with metformin increased compared with individuals without diabetes [181] (Table 2).

MTOR is a multifunctional protein that participates in many signaling pathways including growth factors, energy status, nutrient utilization, and various stressors [182]. Much of the literature suggests that the genetic regulation of mTOR signal transduction can slow the aging of many organisms [183–185]. These signal modulations, including mRNA translation, transcription, autophagy, and mitochondrial function, have been shown to mediate extended lifespan [186]. Moreover, the rapamycin-FKBP12 binding event caused mTORC1 to destabilize, thereby inhibiting mTORC1. As an effective anticancer drug, rapamycin can slow or reverse many age-related changes [187–189] (Table 2). Studies have provided evidence that rapamycin only prolongs lifespan through antitumor mechanisms, thereby inhibiting the main pathological changes in mice [190, 191]. Rapamycin can reverse the increased SASP of senescent cells [192–194] (Table 2). At present, research on the mTORC1 pathway has the strongest clinical evidence, proving that it can be used as a feasible strategy to prevent aging.

NAD^+^ is a coenzyme that catalyzes a wide range of cellular metabolic functions through cellular redox reactions and is converted into NADH through it. Much of the literature suggests that NAD^+^ declines in many tissues during the aging process, including adipose tissue, skeletal muscle, brain, liver, pancreas, heart, spleen, kidney, and lungs, leading to various age-related pathophysiology developments [195–197]. However, NAD^+^ is not absorbed by cells, making direct supplementation impossible. Nonetheless, NAD^+^ supplements have a protective effect during aging, and nicotinamide is the main precursor in NAD^+^ biosynthesis. NAMPT catalyzes the conversion of nicotinamide and 5′-phosphoribose pyrophosphate to nicotinamide mononucleotide (NMN) [198]. NMN can also be synthesized by the NR kinase NRK1 through another NAD^+^ intermediate, nicotinamide ribose (NR). Thus, NMN and ATP are converted into NAD^+^ by NMN adenylate transferase NMNAT1-3. According to reports, NR and NMN have a protective effect in a number of age-related disease models, by increasing the pool of precursors and thus NAD^+^ levels in the body, both of which have been tested in murine and invertebrate aging studies [195, 197, 199]. NR also increases the lifespan of yeast replication [200], and both NR and NMN increase the lifespan of worms [201]. In mice, NR causes a wide range of beneficial effects, including a moderate lifespan extension [202]. These studies also documented that NMN has a significant effect on improving disease conditions and reducing age-related physiological decline [203–206]. NMN treatment can also restore glucose-stimulated insulin secretion in elderly mice [207]. Previous studies have demonstrated that NMN can effectively alleviate age-related physiological decline in wild-type mice fed with conventional feed [205]: through a transporter called slc12a8, 26-month-old mice (equivalent to 70 human years) that took NMN increased their NAD^+^ levels to close to 3 months old (equivalent to 12 human months). The content level in young mice increased the cell energy in aged mice [208]. In general, these findings strongly indicate that NMN and NR are key endogenous compounds in NAD^+^ biosynthesis and can be used as effective treatments to prevent many age-related diseases.

Our understanding of the molecular mechanisms that lead to aging has rapidly increased, creating new opportunities for intervention in the aging process. These studies have yielded two notable findings. First, the number of genes that can extend lifespan is much higher than expected, which indicates that the level of plasticity during aging is highly. Second, genes that control aging are highly conserved in humans. These pathways are conserved over a wide range of evolutionary distances, and targeting these pathways in model organisms prolongs lifespan. Together, these evidences indicate that the mechanisms that interfere with aging may have great implications.

## Conclusion and perspectives

Overall, immune parameters are different between the young and the elderly. Indeed, the aging process particularly affects the immune response: this imbalance of the immune system may be involved in the development of tumors. Currently, there are relatively few systematic studies on immunosenescence. Therefore, understanding the level of age-related immune changes requires a systematic approach to studying the multiple interacting factors of immune response. As indicated above, immunosenescence is a complicated and inevitable process that seems to affect tumor progression and the efficiency of antitumor immune responses in the elderly individuals.

The impact of immunosenescence on tumor progression indicates that it is necessary to better understand its role in treating and driving tumor progression. First, the reduction of T cell output caused by thymic degeneration can be achieved through the intervention with immune regulatory factors, i.e., by supplementing IL-7 [30], thereby restoring T cell production and thymic function. Furthermore, the rejuvenation strategy for the thymic matrix microenvironment has been proven to successfully restore the thymus function of the elderly. Second, by intervening the SASP during the aging, combined therapy can be used to achieve better therapeutic effects. For example, the SASP induced by chemotherapy increases the sensitivity of PD-1 and chemotherapy, thereby reducing tumor progression and metastasis [78]. Finally, emerging evidence suggests that the aging microenvironment largely contributes to age-related decline in immune function and may also provide potential targets for rejuvenating the immune system [18].

Moreover, although basic and clinical research on aging has made great progress, the existing immunological techniques cannot thoroughly analyze the complexity of the immune system. For example, the 6–8-week mouse model is the most frequent model employed in tumor studies; thus, the model may not be entirely applicable to humans [209, 210]. Furthermore, the definition of the corresponding age between humans and mice is still under discussion [210, 211], and a more convincing in vivo aging model between the age of immunity and time series should be established [211]. Therefore, a more profound understanding of immunosenescence, especially of its underlying mechanisms and therapeutic targets in malignant tumors, is urgently needed to reduce this growing public health burden. In the future, targeting immune senescent cells may be a novel interventional opportunity in cancer patients.

## Data Availability

Not applicable.

## Abbreviations

cAMP: Cyclic adenosine monophosphate
CAR-T: Chimeric antigen receptor
CTLA-4: Cytotoxic T-lymphocyte-associated protein-4
ECM: Extracellular matrix
ICB: Immune checkpoint blocking
IFN-γ: Interferon gamma
IL-7: Interleukin-7
KLRG-1: Killer cell lectin-like receptor subfamily G
MDSC: Myeloid-derived suppressor cells
NK: Natural killer
NMN: Nicotinamide mononucleotide
NR: Nicotinamide ribose
PD-1: Programmed cell death protein
PD-L1: Programmed cell death 1 ligand 1
ROS: Reactive oxygen species
SASP: Senescence-associated secretory phenotype
TME: Tumor microenvironment
Tregs: Regulatory T cells
TSCM: Stem cell memory T cells
