# Correction: Immunosenescence: a key player in cancer development

**DOI:** 10.1186/s13045-025-01682-6

**Published:** 2025-03-05

**Authors:** Jingyao Lian, Ying Yue, Weina Yu, Yi Zhang

**Affiliations:** 1 Biotherapy Center and Cancer Center, The First Affiliated Hospital of Zhengzhou University, 1 Jianshe East Road, Zhengzhou, 450052 Henan China; 2 State Key Laboratory of Esophageal Cancer Prevention and Treatment, Zhengzhou, 450052 Henan China; 3 Clinical Laboratory, Henan Medical College Hospital Workers, Zhengzhou, 450000 Henan China

**Correction: Journal of Hematology & Oncology (2020) 13:151**

10.1186/s13045-020-00986-z

In the ahead-mentioned sentence in the original article, ‘KLRG1' was incorrectly written as ‘NKG2C’, leading to an inaccurate statement.

The authors regret this oversight and wish to clarify that the corrected sentence should instead read:

“It should be noted that, in the process of immunosenescence, the activating receptor expression of natural killer (NK) cell markers NKP30, NKP46, etc., is reduced, while the inhibitory receptor expression of KIR, KLRG1, etc., is increased”.

